# Active Colloidal Molecules with Dynamic Configurational Freedom

**DOI:** 10.1021/acsnano.5c07142

**Published:** 2025-08-11

**Authors:** Stefania Ketzetzi, Lorenzo Caprini, Vivien Willems, Laura Alvarez, Hartmut Löwen, Lucio Isa

**Affiliations:** † Laboratory for Soft Materials and Interfaces, Department of Materials, ETH Zürich, 8093 Zürich, Switzerland; ‡ Institut für Theoretische Physik II: Weiche Materie, Heinrich-Heine-Universität Düsseldorf, D-40225 Düsseldorf, Germany

**Keywords:** micromachines, microrobots, microswimmers, micromotors, self-assembly, active soft matter

## Abstract

Molecular machines and microorganisms employ dynamic shape changes to enable adaptive function. In contrast, active colloidal machines and micromotors, their synthetic counterparts, are typically preconfigured and mechanically rigid, which limits the range of their dynamic behavior and thereby their functionality. Here, through physical interactions alone, we assemble active colloidal molecules with flexible configurations that evolve freely and continuously in time. Unlike existing colloidal systems that either offer structural flexibility in passively diffusing assemblies, or impose fixed configurations in self-propelling ones, our colloidal molecules both dynamically self-assemble and disassemble on demand and directly propel themselves through their own internal restructuring. This, in turn, bestows enhanced self-regulation, self-steering, and avoiding capabilities upon encountering other molecules. These capabilities suppress clustering and motility-induced phase separation, allowing them to remain dispersed, well-separated, and still actively moving even at high concentrations. Micromotors with dynamic configurational freedom thus constitute a step toward autonomous motion beyond classical synthetic active matter, and allow for designing “intelligent” microrobots and responsive functional active materials at the nano- and microscale.

## Graphical abstract

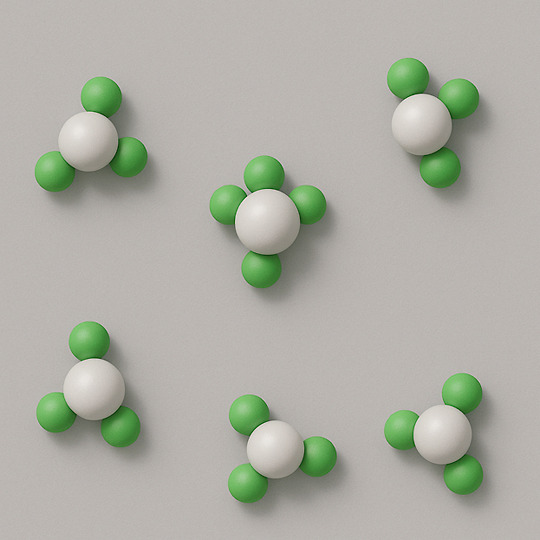

## Introduction

The design of microscale machines has long been inspired by nature’s ability to dynamically assemble, disassemble, and reconfigure functional structures in response to environmental cues. In biological systems, molecular machines such as motor proteins and multienzyme complexes continuously reorganize their structure to perform complex tasks under nonequilibrium conditions.^1–7^ These dynamic processes are key for adaptability, efficient energy conversion, and robust function.

In contrast, many synthetic approaches to create colloidal analogues have so far been limited, with full structural flexibility achieved only in passive colloidal molecule assemblies.^8,9^ For instance, flexible colloidal joints assembled via surface-mobile DNA linkers enable designed motion ranges and serve as building blocks for reconfigurable architectures, approximating the “soft” degrees of freedom of molecular systems.^10,11^ However, such systems lack self-propulsion and autonomous driving.

Synthetic micromotors, otherwise known as artificial microswimmers or active colloidal particles, are often proposed as minimal models displaying physical behavior inspired by biological systems across length scales due to their self-propulsion capabilities.^12–16^ However, to date, dynamic reconfiguration escapes experimental realization in such systems. Self-propelled colloids typically comprise preconfigured and mechanically rigid units, which inevitably couple translation and rotation through thermal fluctuations.^13^ Recently, colloidal constructs self-propelling under external fields have been engineered to switch between discrete dynamical modes induced by cues such as temperature or light.^17–20^ However, these too have predetermined states controlled by the geometry and lack full configurational freedom.

A lack of flexibility in structure as well as motion on the level of individual micromotors can also result in limited modes of self-organization on the collective level. A constant self-propulsion velocity and motion persistence, together with excluded volume interactions, lead to dynamic clustering, motility-induced phase separation, and other types of coordinated organizations, such as swarms, chains, vortices and flocking.^21–40^ Consequently, current realizations cannot completely reproduce the fully autonomous behaviors typical of biological units. Incorporating internal flexibility through dynamic reconfiguration in synthetic active matter may thus allow novel, fully flexible modes of motion and organization.

Here, we realize fuel-free micromotors in the form of active colloidal molecules that self-assemble, move, and fully reconfigure dynamically through physical interactions alone. We find that upon encountering neighboring molecules, dynamically reconfiguring molecules steer their direction of motion and actively avoid one another via effective antialignment interactions. Furthermore, reconfiguration-induced avoidance fully prevents the aforementioned dynamic clustering, motility-induced phase separation and flocking observed in rigid active particles, and instead enables them to remain dispersed and well-separated. Through experiments and simulations, we show that dynamic configurational freedom decouples the reorientation of our colloidal molecules from their rotational diffusivity, shifting the time scale of motion persistence to that of internal restructuring. This behavior—distinct from that of traditional, mechanically preconfigured active colloids—enables a new regime of control over self-propelled motion and is particularly desirable for designing “intelligent” micromotors and exploring complex biological behaviors using synthetic particles.^41–44^

## Results and Discussion

### Self-Reconfiguring Active Molecules

We trigger the formation of active colloidal molecules using an alternating current (AC) electric field^18,45–53^ and further demonstrate that they exhibit dynamic configurational freedom that leads to self-propulsion stemming from purely physical interactions between their constituents. We mix aqueous suspensions of silica (SiO_2_) and fluorescently labeled polystyrene (PS) colloids that differ in size and dielectric properties (Experimental Section and Supporting Information). The SiO_2_ and PS radii are *R*_c_ = 2.8 μm and *R*_s_ = 0.35 μm, respectively (see Supporting Information for a discussion on the effect of size ratio on molecule assembly and dynamic configurational freedom, and Figure S1 for another example of assembly of molecules using differently sized spheres under the same field conditions). We confine the suspensions between two transparent conductive surfaces separated by a spacer (thickness 2H = 120 μm), and connect the system to a function generator through which we apply a transverse AC electric field of *V*_pp_/2*H*, where *V*_pp_ is the peak-to-peak voltage amplitude. As *H* is fixed in our experiments, we vary and report data as a function of *V*_pp_. We follow all colloids with an inverted microscope at 10 frames per second under simultaneous bright-field and fluorescence illumination.

In the absence of the field, colloids sediment and diffuse near the bottom surface (Figure 1a). At frequency 1 kHz, SiO_2_ colloids effectively act as dipoles that distort the electric double layer along the electrode, creating electrohydrodynamic (EHD) flows.^54^ These flows point inward in the direction perpendicular to the electrode attracting the PS colloids in their vicinity (Figure 1b–e), see also Figure S2 for detailed calculations based on EHD theory.^18^ In brief, SiO_2_ and PS particles drive EHD flows of opposite symmetry under our experimental conditions, with the former generating contractile flows—fluid is drawn inward along the substrate toward the particle—and the latter producing extensile flows—fluid is pushed outward along the substrate. This difference stems from their dielectric properties: at low field frequencies, the SiO_2_ is less polarizable than the surrounding medium, while the PS shows the opposite. Under our chosen conditions, the asymmetric EHD flow generated by a combined SiO_2_–PS pair drives both their assembly and propulsion.

**Figure 1:**
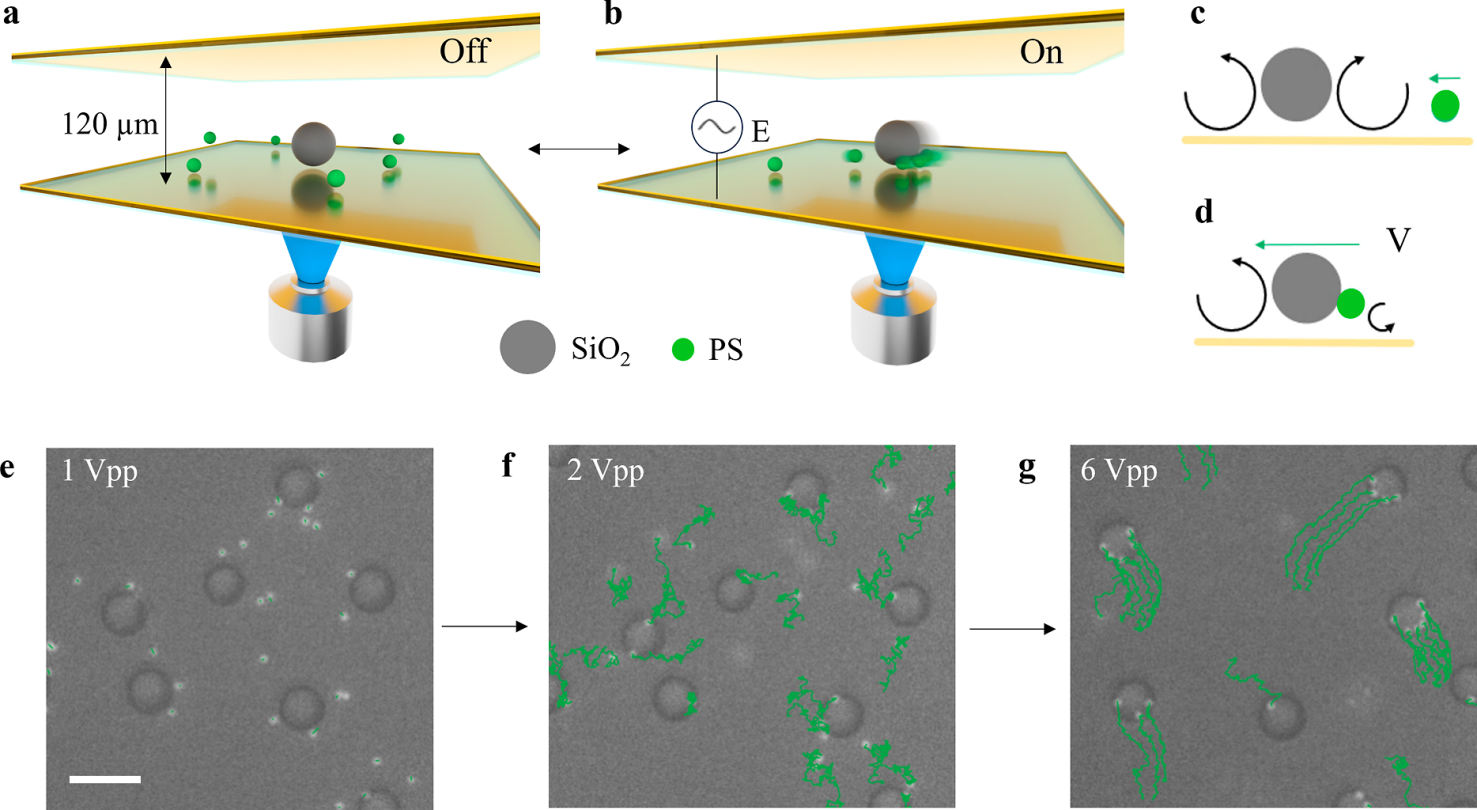
Self-reconfiguring active colloidal molecules. (a,b) Schematic representation of the experimental system. (a) SiO_2_ and polystyrene (PS) colloids suspended in water are confined between two transparent conductive surfaces for observation with an inverted microscope. (b) Under a perpendicular AC electric field, colloids self-assemble into self-reconfiguring active molecules. (c,d) Schematics of the assembly and propulsion mechanism. (c) At a frequency of 1 kHz, electrohydrodynamic flows along the SiO_2_ surfaces attract PS particles in their vicinity. (d) Upon increasing AC voltage peak-to-peak amplitude (*V*_pp_), PS colloids assemble around the SiO_2_, causing an asymmetry in the fluid flow that propels the molecules forward. (e–g) Optical micrographs depicting molecule self-assembly and active motion. The PS particles and their trajectories are shown in green. (e) At 1 *V*_pp_, PS colloids approach the SiO_2_. (f) At 2 *V*_pp_, PS colloids (satellites) assemble around the SiO_2_ (core) forming molecules. The PS satellites continuously translate along the cores’ surface. (g) At 6 *V*_pp_, the self-assembled molecules exhibit active self-propulsion. Scale bar is 10 μm.

Upon increasing the field amplitude *V*_pp_, nearby PS colloids assemble onto the SiO_2_ surfaces forming colloidal molecules, with the SiO_2_ colloids acting as the core of the molecules and the PS particles behaving as their satellites (Figure 1f and refs 49,50 and 55 for details on field-driven assembly). Upon assembly, PS colloids break the initial radial symmetry of the flow around their SiO_2_ core, thus generating an asymmetric flow that self-propels the entire molecule with the PS colloids at its back (Figure 1g). For the full process, including molecule self-assembly and propulsion under the electric field and with increasing *V*_pp_, see Video S1.

Crucially, here the PS colloids “bind” flexibly and are free to orbit around the SiO_2_ core: driven by Brownian fluctuations, satellites continuously undergo translational diffusion along an orbit slightly below the core’s equatorial plane. To pinpoint the effect of dynamic reconfiguration on active motion, we also preassemble molecules with fixed configurations using the same set of colloids (Experimental Section and Figure S3) but permanently attaching the PS colloids onto the SiO_2_ particles.

### Molecules with Minimal Composition

First, we examine dimers, comprising a core and one satellite, as a minimal system. We track positions and quantify active motion through standard particle tracking algorithms,^56^ see Figure S3 for the average speed *V* = |Δ*r⃗*|/Δ*t* of each core and hence molecule, with Δ*t* = 0.1 s set by the frame rate, as well as the configuration of the molecule with respect to the electrode obtained directly from the particle positions and from the satellite-core separation distance. Our measurements agree with EHD theory calculations that account for system-specific parameters (Supporting Information).

We quantify the orientational dynamics of the reconfiguring molecules by tracking the orientation angle θ of the velocity vector between frames. We extract the reorientation time τ_R_ of each molecule by fitting its mean square angular displacement (MSAD) ⟨Δθ­(*t*)^2^⟩ = ⟨|θ­(*t* + *t*_0_) – θ­(*t*_0_)|^2^⟩ as a function of time with the expression ⟨Δθ­(*t*)^2^⟩ = 2*t*/τ_R_ (Figure S3). We present the values of τ_R_ as a function of field amplitude for our dynamically self-reconfiguring molecules in Figure 2a (error bars not visible in logarithmic scale). In the same figure, we also present values obtained from control experiments using preassembled molecules with a fixed PS particle.

**Figure 2:**
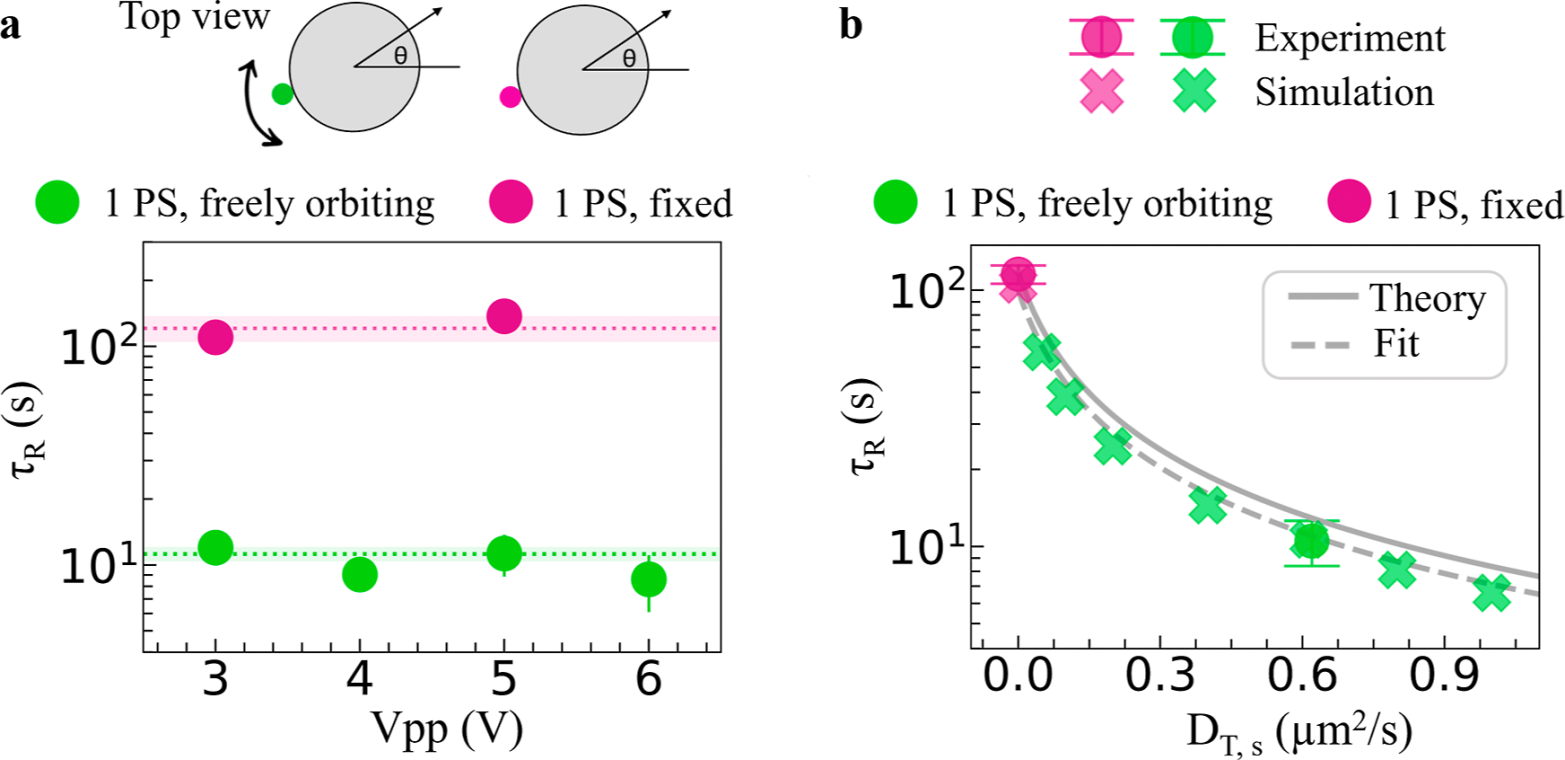
Decoupling reorientation from rotational diffusivity. (a) Time scale of reorientation τ_R_ as a function of peak-to-peak amplitude *V*_pp_ measured for both reconfiguring and preassembled dimers. Dotted lines show mean values, shaded areas standard deviations. The schematic shows a top view of the dimers, with θ representing the orientation of the velocity vector. b) Time scale of reorientation as a function of satellite translational diffusivity *D*_T,s_ from simulations and experiments; errors represent standard errors.

Strikingly, the measured time scales for reorientation differ by an order of magnitude, irrespective of field amplitude, even though both dynamically reconfiguring and preassembled objects have essentially the same dimensions and move under the same conditions. We find that the time scale for the control, preassembled molecules τ_R_ = (121 ± 16) s agrees well with the prediction for the time scale of rotation of the core *t*_R,c_ = 1/*D*_R,c_ = 135 s given by the Stokes–Einstein equation for rotational diffusion, even including small corrections due to presence of the wall (*D*_R,c_ = *k*_B_ *T*/8πη*R*_c_^3^ with *k*_B_ the Boltzmann constant, *R*_c_ the core radius, η and *T* the shear viscosity and temperature of the bath, respectively). We recover the same value for the case of two fixed PS particles (Figure S3). At the same time, the τ_R_ = (11 ± 1) s measured for the dynamically reconfiguring molecules is much faster than the Brownian rotation of the core particle. The measured reorientation time instead agrees well with the time scale for one-dimensional satellite diffusion *t*_T,s_ ∝ ρ^2^/*D*_T,s_ = 10 s, as obtained from the Stokes–Einstein equation for translational diffusion *D*_T,s_ = *k*_B_ *T*/6πη*R*_s_ assuming a particle of the same size as the satellite orbiting a circular path, with ρ the orbit radius from the measured satellite-core distance.

### Modeling

To verify the leading mechanisms that couple the self-reconfiguration of a molecule to its reorientation, we describe satellite and core particles with the same size ratio as in the experiments by two-dimensional diffusive objects with dynamics satisfying the Einstein relations. In brief, when a satellite, by diffusion, approaches a core particle closer than a cutoff distance, strong attractive interactions lead to the formation of a molecule, which breaks translational symmetry causing active motion. The asymmetric flow around the molecule, modeled by an effective force, induces an active velocity with direction along the line joining the centers of the satellite and core.

With these minimal ingredients, the satellite can diffuse around the core and its angular position determines the direction of the propulsion velocity of the whole molecule, as in the experiments. As a result, the present model goes beyond standard active Brownian particle dynamics, where particle orientation would only be governed by the rotational diffusion of the whole molecule as a rigid body. Orientation is here intrinsically controlled by the instantaneous configuration of the molecule driven by satellite diffusivity, see the Supporting Information for details on the model.

Our model quantitatively reproduces the time scale for reorientation measured experimentally in both the dynamically reconfiguring and preassembled cases (Figure 2b), and predicts a systematic dependence of τ_R_ of the satellite’s diffusivity beyond the experimental realizations (Figure S4). In particular, we verify the hypothesis that τ_R_ decreases as the satellite translational diffusion *D*_T,s_ increases, i.e. that the time scale for reorientation is mainly determined by the diffusive motion of the satellite, which induces the molecule’s self-reconfiguration and orientational freedom. Since *D*_T,s_ can be controlled via tuning the satellite size according to the Einstein relation, this finding is highly relevant for designing complex molecules with controllable reorientation dynamics.

### Molecules with Complex Composition

To unravel further implications of dynamic reconfiguration on active motion, we then examine complex colloidal molecules with two or more satellite particles as in Video S2. Figure 3a shows a measured time series of a self-reconfiguring active trimer with two satellites. We observe that, as satellites diffuse around the core, they continuously probe different instantaneous configurations.

**Figure 3:**
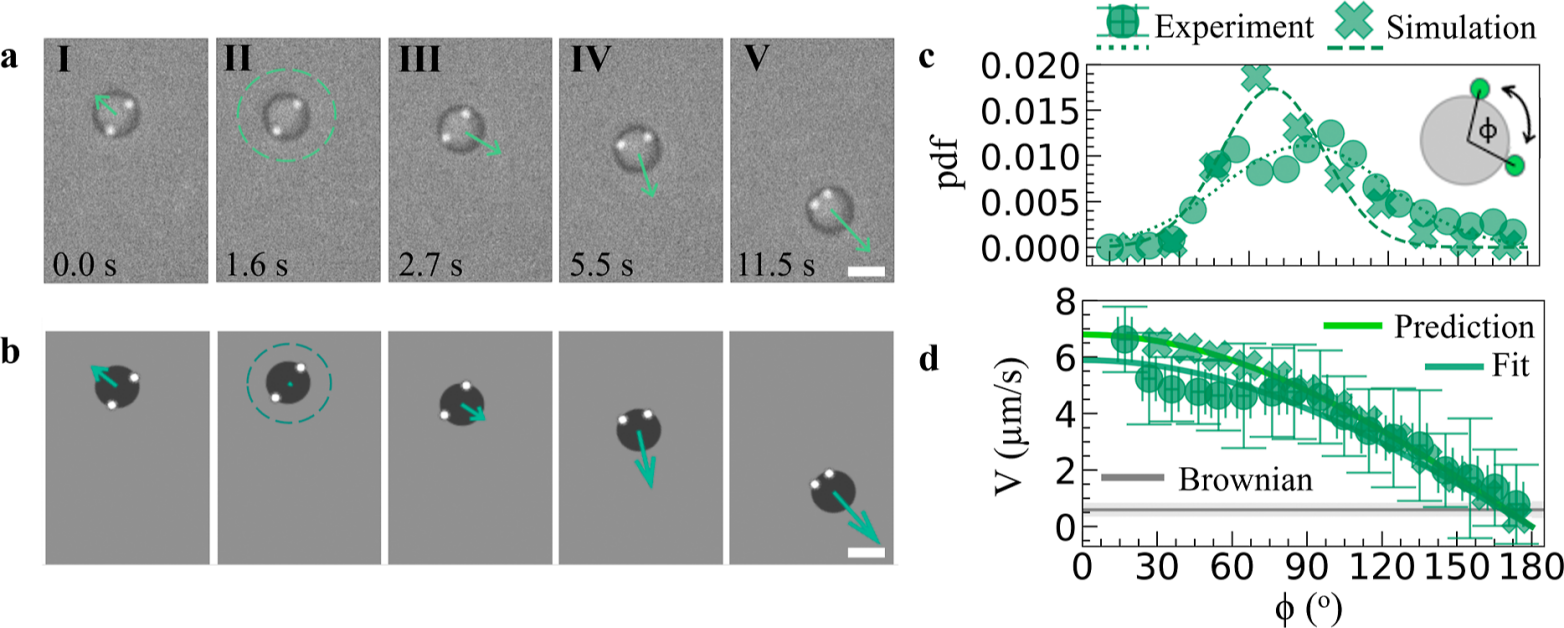
Reconfiguration-induced reorientation at the single-molecule level. (a,b) Time series of a dynamically reconfiguring active trimer from (a) experiments and (b) simulations; arrows indicate the magnitude of the velocity. Over time, satellites probe different configurations, resulting in directed motion—when they are distributed asymmetrically with respect to the core—or a stationary state—for symmetric configuration—followed here by reversal of the motion direction when both satellites cross over to one side of the core (scale bar 5 μm). (c) Probability density function of the instantaneous opening angle ϕ between satellites. Dotted and dashed lines are fitted Gaussian distributions. (d) Active velocity of reconfiguring trimers as a function of ϕ in experiments and simulations. The active velocity decreases with opening angle following a simple geometric argument as presented in the text; errors are calculated from standard deviations as described in the text. The gray line marked as ”Brownian” corresponds to the mean displacement over 0.1s for passive particles.

Satellites can be distributed either asymmetrically relative to the core (column I) or symmetrically around it (column II). The asymmetric configuration leads to symmetry breaking of the EHD flows, and correspondingly to self-propulsion with the satellites at the back, as for molecules with a single satellite. The symmetric configuration leads to a radially symmetric fluid flow and an instantaneous stationary state, with near-zero net propulsion force. After going through a symmetric configuration, a change in the net direction of motion can be observed (column III) and the molecule self-propels in a new direction defined by the subsequent asymmetric configurations (columns IV and V). This is a representative realization of a reorientation event, on noting that due to the randomness of satellite diffusion, in another instance the motion direction could have been retained after a symmetric configuration. We reproduce the motion of reconfiguring trimers in our simulations imposing that each satellite provides an active force and contributes to determining the total molecule dynamics (Figure 3b). We recover the experimentally observed configurational changes by introducing an effective long-range repulsion between satellites, effectively induced by hydrodynamic interactions. Note that the soft angular confinement resulting from repulsion and steric effects does not create directional bias since the satellites are free to orbit around the core. Importantly, the absence of prescribed geometrical or chemical bonds in our system makes the reconfiguration predominantly stochastic. The overall reorientation and reversal behavior is governed by satellite Brownian diffusion rather than a deterministic switching mechanism.

Qualitatively, the instantaneous configuration of the satellites affects self-propulsion both in the experiments and simulations. We subsequently quantify the continuously fluctuating opening angle ϕ between satellites in the experiments and correlate it to the magnitude of the active velocity. The probability density function of opening angles obtained from different trimers with two satellites shows a bell-shaped distribution which resembles a Gaussian (Figure 3c), commensurate with the Brownian nature of the satellites’ motion, with a peak value ϕ_P,Exp_ = (84 ± 36)° determined by a least-squares fit (dotted line). The experimental behavior is well captured by our simulations, with ϕ_P,Sim_ = (70 ± 22)°. Once the instantaneous configuration of the molecules is known, the corresponding instantaneous active velocity is determined. We bin the data by opening angle (bin width = 10*°*, field frequency 1 kHz, amplitude 6 *V*_pp_) and fit the velocity and angle distributions within each bin with a Gaussian; we report the fitted means and corresponding standard deviations in Figure 3d. For reference, the average Brownian “speed” of a single core particle *V*_0_ = (0.6 ± 0.3) μm/s, obtained from its Brownian displacements at Δ*t* = 0.1 s, is also shown in gray.

Assuming that each satellite contributes equally to the active force by the corresponding EHD flows, we predict that the velocity would follow the “sum rule” expression *V*_pred_ = 2 *V*_1_cos­(ϕ/2), with *V*_1_ corresponding to the experimental velocity of dynamically reconfiguring dimers at the same field condition in Figure S3 (*V*_1_ = (3.4 ± 0.7) μm/s, error denotes standard error). We show that this geometrical sum accurately describes our measurements in Figure 3d (green line “Prediction”). Fitting the data while keeping *V*_1_ as an open fit parameter yields *V*_fit_= (3.0 ± 0.1) μm/s (green line “Fit”, error obtained from the covariance matrix), in line with the measured *V*_1_. Simulations reproduce this behavior in Figure 3d. Note that while the “initial” instantaneous configuration defines the instantaneous speed, it does not determine long-term directionality. Therefore, while a deterministic feature such as the initial opening angle can affect transient propulsion strength, our observations emerge from stochastic configurational dynamics as per our previous discussion.

We confirm that the sum rule of the velocity also holds for different numbers of satellites, see Figure S5 for data on tetramers with three satellites. Note that the number of satellites and thus possible accessible configurations plays a role on whether the molecule can retain asymmetry with respect to the core and move faster without reversal. Molecules with fewer satellite particles effectively reorient more slowly (Figure S4). In this case, the configuration can remain imbalanced for longer, yielding sustained motion. Conversely higher satellite numbers “fill in” the core perimeter, leading to slower average motion and more frequent reorientation. At full valency, no net directed motion is observed, as expected by symmetry. Together, our findings suggest that manipulating both the molecule composition and configuration would allow tailoring the active velocity.

### Dynamic Configurational Freedom Induces Self-Steering and Avoidance

We next turn to the implications of self-reconfiguration at the level of the interactions between active molecules, seeking to understand the role of reconfiguration-induced reorientation on self-organization at the collective level. Strikingly, when two of our active molecules approach, they sense each other’s presence at a distance and respond by changing propulsion direction through spontaneous reconfiguration (Video S3). The process is exemplified in Figure 4a-I.

**Figure 4:**
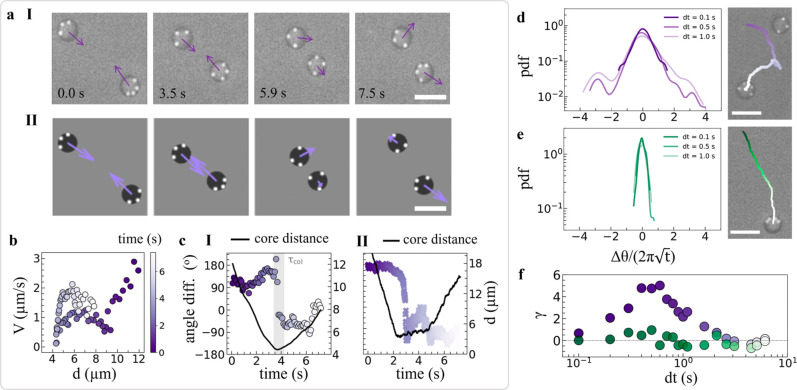
Reconfiguration-induced avoidance. (a) Time series of interacting molecules from (I) the experiments and (II) simulations; arrows indicate the direction and magnitude of instantaneous velocities. (b) Velocity of a dynamically reconfiguring pentamer with four satellites as a function of distance from its neighbor. Time is color-coded according to the vertical bar. (c) Angle difference between the orientation of the velocity of interacting molecules as a function of time measured in (I) experiments and (II) simulations. The right axis shows the intermolecular distance, with the shaded region highlighting the duration of the “collision”. Distribution of angular displacements of the core of a self-reconfiguring pentamer at different lag-times and corresponding active trajectory (d) in the presence of interactions and (e) without interactions. (f) Kurtosis of the angular displacement distributions at different lag-times with and without interactions with neighbors; scale bars are 10 μm.

Initially, the molecules self-propel toward each other with the satellites at the back. However, upon coming within a critical separation distance, the respective satellites experience a long-ranged attraction to the neighboring core and correspondingly reconfigure, seemingly acting as sensors. With decreasing distance, repulsion between the cores takes over and prevents contact between molecules or satellite exchange. Nonetheless, at this stage, the satellites have migrated to the other side of the core, thus inducing a reversal of propulsion direction, leading the particles to self-steer away from each other. This change in direction happens much faster (≲1 s) than the previously discussed self-reconfiguration-driven reorientation without interactions, while it is still mediated by satellite reconfiguration. In essence, interacting molecules actively exhibit reconfiguration-induced avoidance, reminiscent of self-avoidance observed in motile organisms across length scales and different from avoidance due to pure repulsion as we discuss in the following section.

We quantify the different stages of self-avoiding interactions by measuring the velocity of one of the two molecules (the pentamer in Figure 4a­(I) in Figure 4b, and the angle between the velocity vectors of the two molecules as a function of intermolecule distance in Figure 4c­(I). Upon approach, at large separation distance (color-coded in the side bar as a function of elapsed time), the velocity decreases as the molecule’s satellites move toward the other side of the core via configurations that are more symmetric with respect to the center, which correspondingly slow down the molecule in line with the results of Figure 3. Repulsion between cores, mediated by electro-hydrodynamic flows,^57^ additionally contributes to hindering the forward motion of the molecule. The velocity goes to zero at a minimum distance (≈4.5 μm), at which the satellites become tightly packed at the opposite hemisphere, pushing the molecules in the opposite direction. As the distance increases, satellites cease sensing the presence of a neighboring particle and resume Brownian diffusion along their core. The reversal in the sign of the velocity vectors at a minimum distance in Figure 4c­(I) clearly indicates antialignment (note that, for convention, we define positive and negative angles as the aligned and antialigned states, respectively).

However, since the direction reversal happens faster than natural reorientation, these antialigning interactions affect the distribution of angular displacements in the propulsion direction differently at different lag-times dt (Figure 4d). In particular, at time scales shorter than the particle reconfiguration, e.g. d*t* = 0.1 s, the angular displacements follow a Gaussian distribution centered around zero, as for active Brownian particles, whose width is defined by the satellites’ *D*_T,s_ as initially discussed. On the other hand, at lag-times comparable to the characteristic duration of “collisions”, large angular-displacement tails emerge (d*t* = 0.5 s and d*t* = 1.0 s). As a comparison, the same analysis on a self-reconfiguring pentamer that does not interact with any other molecule along its active trajectory shows no sign of non-Gaussian behavior (Figure 4e). The evolution of non-Gaussian orientational dynamics at different lag-times is readily quantified by the kurtosis γ of the distributions of angular displacements, plotted in Figure 4f; γ­(d*t*) > 0 corresponds to heavy tails and γ­(d*t*) = 0 to a Gaussian distribution. We observe that the kurtosis peaks at 0.6 s, comparable to the collision time, and that this behavior persists across different molecules with varying compositions undergoing multiple collisions (Figure S6).

We furthermore fully reproduce this behavior in simulations (see Figure 4a­(II),c­(II) and Supporting Information for details on modeling interactions) confirming that the interplay between the molecule reconfiguration and satellites–core interactions are responsible for effective antialignment interactions. This confirmation additionally corroborates the mechanism underpinning reconfiguration-induced avoidance in our system and in turn determines minimal ingredients for its realization.

### Flexible Organization at the Collective Level

Dynamic reconfiguration here decouples reorientation from rotational properties and sets the instantaneous velocity leading to speed modulation and reorientation on the single-molecule level, such that translational and rotational motions are effectively independent. Thus, active molecules with dynamic configurational freedom are governed by distinctly different dynamics compared to traditional active colloids.

Standard active colloids with a constant velocity and fixed rotational diffusivity phase separate or form dynamic clusters at area fractions as low as ≈3%,^23,24,26^ while our reconfiguring molecules remain well-separated up to the highest investigated area fractions of ≈16% (examples in Figure 5 and Video S4). Intriguingly, at the collective level the qualitative dynamics of the system does not appear to be strongly affected by the number of satellites of the active molecules, as observed by comparing the experiments to the monodisperse case of active tetramers with three satellite particles from the simulations (Figure 5b). We numerically check whether antialignment mechanisms are also present at the collective level. Indeed, we find that the distribution of the angle formed by the active speed vector, which is solely determined by the molecule configuration, and the velocity for each particle displays non-Gaussian tails reaching values close to π and −π in Figure 5c. Their presence suggests that reorientation events caused by interactions take place before a molecule starts moving in the direction determined by its active speed. That is, two particles have a probability of moving in the opposite direction compared to the particle orientation, as it is the case during reorientation events. After reorientation, the particle moves again in the same direction of the active speed vector. For reference, the dotted line in the same figure shows a Gaussian distribution.

**Figure 5:**
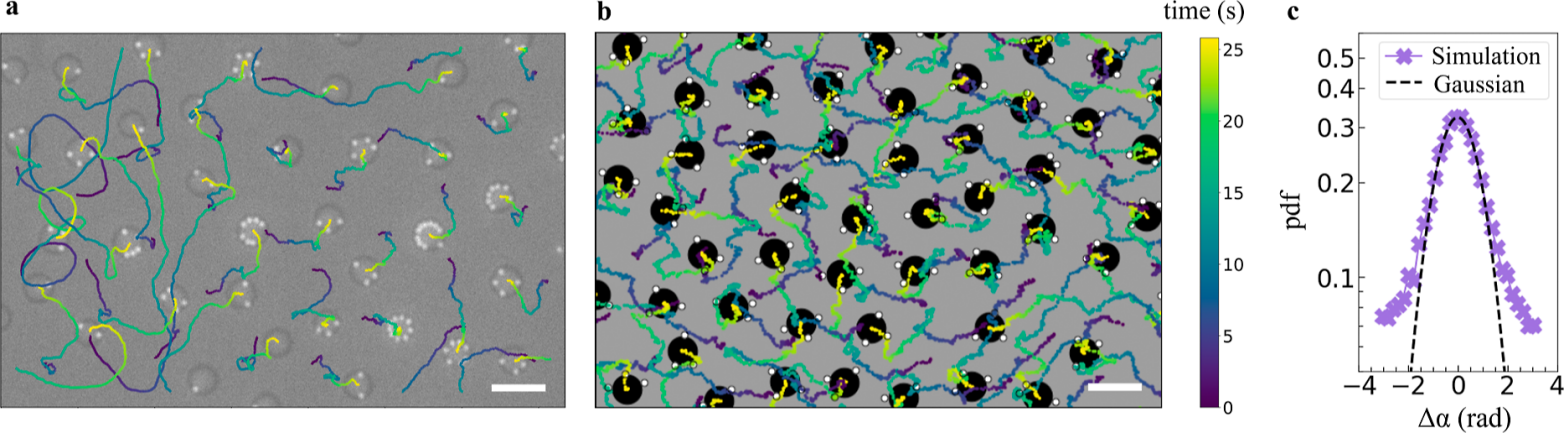
Collective dynamics: reconfiguration-induced avoidance hinders activity-induced clustering, phase separation and flocking at high area fractions, different from traditional active colloids. Self-reconfiguring molecules uniformly occupy space while allowing for collective rearrangements without activity-induced clustering in both (a) experiments, with molecules of varying compositions, and (b) simulations of tetramers with three satellites. Area fractions are 0.11 and 0.16, respectively. All lines represent trajectories color-coded with time. Scale bars are 10 μm. (c) Probability distribution of the angle difference between the active speed vector determined by the molecule configuration and the velocity vector as obtained from the simulations, showing non-Gaussian tails. Dotted black line shows a Gaussian distribution.

Reconfiguration-induced avoidance here promotes a uniform spatial distribution of the particles while maintaining fluidity and allowing for collective rearrangements driven by activity. This active type of avoidance differs from avoidance due to repulsion alone observed recently in rigid active colloids which led to flocking and formation of active Wigner crystals.^39^ Our system thus constitutes a promising candidate for studying collective dynamics in active glassy systems,^58^ or for constructing hyperuniform systems.^42,59^ In particular, reconfiguration-induced avoidance tends to homogenize particle distribution such that density fluctuations are strongly suppressed, which is a prerequisite for hyperuniformity.^60^

Finally, the type of antialignment after collisions that we report here is in contrast with the requirements to establish the classical, previously observed motility-induced phase separation, as well as typical flocking. In those cases, alignment promotes the transition, even in the case of repulsive interactions.^39^ Antialigning interactions can conversely induce completely different patterns, as recently explored by theoretical studies,^61–63^ including dancing hexagons and exotic wavy patterns. Reconfiguration-induced antialignment has insofar remained unexplored, noting that in previous studies^64,65^ single particles changed their orientation relative to the propulsion direction after experiencing a torque.

## Conclusions

Synthetic micromotors, although highly promising as minimal models for motile systems, are typically fabricated as mechanically rigid units with fixed configurations. Without the ability to internally reconfigure their structure, these systems inevitably couple translation and rotation through thermal fluctuations, leading to phenomena such as dynamic clustering, motility-induced phase separation, and flocking.

We instead realize active colloidal molecules with flexible configurations that continuously evolve in time. First, the structures can assemble and disassemble on demand. Second, once assembled, the relative positions of the constituent units are free to fluctuate allowing for dynamic configurational freedom. This results in a flexible colloidal machine that creates its own propulsion through continuous configurational changes, and might therefore serve as a prototype for mimicking adaptive biological behavior and organization while retaining robust self-propulsion.

Remarkably, dynamic restructuring decouples the reorientation dynamics from the rotational diffusivity, endowing our molecules with enhanced self-steering capabilities that enable directional adjustments during encounters with neighbors. This dynamic reconfiguration, which integrates active and directed motion, provides a novel platform for exploring nonequilibrium behavior in meso- and nanoscale assemblies and allows for designing microrobotic systems and responsive materials with adaptability. By bridging the gap between the flexible but passive colloidal assemblies and the active yet preconfigured systems, our work offers a pathway toward “intelligent” micromotors.

We propose self-reconfiguration as a fundamental mechanism for enabling autonomous steering and avoidance. This capability might be essential for developing particles with advanced navigation capabilities inside complex environments, supporting versatile modes of transport as well as active surface response and representing a key step toward the design of biologically inspired, functional synthetic systems.

## Experimental Section

### Colloidal Molecules

Polystyrene particles with diameter 0.71 ± 0.02 μm (green fluorescent) and silica particles with diameter 5.64 ± 0.27 μm were purchased from Microparticles GmbH. Preassembled molecules used as controls were formed by mixing SiO_2_ and PS particles in Milli-Q water containing 50 mM HCl for 15 min while shaking at 500 rpm at 80 °C (SiO_2_O 0.1 wt %, PS 0.001 wt %), followed by thermal sintering in the oven for 15 min at 90^*o*^C and harvesting with a water droplet into the experimental cell. SEM imaging showed that this process overall did not affect the particles. Self-reconfiguring molecules were self-assembled inside the experimental cell described below in the following manner: SiO_2_ (≈0.005 wt %) and PS (≈0.0005 wt %) particles were mixed in Milli-Q water and placed in the cell, where they sedimented above the bottom electrode. Self-assembly was triggered under the AC electric field for *V*_pp_ > 2 V at a frequency of 1 kHz. The initial particle concentration is not crucial as variations in molecule composition are naturally observed throughout the sample, albeit increasing the PS concentration triggers formation of molecules with full valency that do not display directed motion (cores with ≃ 1 ring of PS).

### Experimental Setup and Imaging

The particle suspension was placed in a custom-made sample cell that consisted of two transparent electrodes separated by an adhesive spacer with a 9 mm-circular opening and 120 μm height (Grace Bio-Laboratories SecureSeal). The electrodes were glass slides (borosilicate glass 18 × 18 mm no 2 corning) coated via electron beam metal evaporation with 3 nm Cr and 10 nm Au (Plassys MEB550S) and plasma enhanced chemical vapor deposition with 10 nm of SiO_2_ (Multiplex CVD, Oxford PlasmaPro 100) to reduce particle adhesion on the conductive glass slide. The electrodes were connected to a function generator (National Instruments Agilent 3352X) that applies the AC electric field (frequency 1 kHz, *V*_pp_ 2–6 V). Particles were imaged using ×20, ×40 and ×63 air objectives (Zeiss) mounted on an inverted microscope (Axio Observer D1) with an sCMOS camera (Andor Zyla) at a frame rate of 10 fps under bright-field transmission illumination in combination with epifluorescence illumination (North 89, filter AF488 nm—excitation: 450–490 nm, emission: 515–1100 nm) to simultaneously identify both SiO_2_ and fluorescently labeled PS particles comprising the molecules. Image sequences were preprocessed on ImageJ and subsequently analyzed using standard particle tracking algorithms in Python or Matlab.

## Supplementary Material











## Notes

Full description of Videos S1–S4; calculation of electrohydrodynamic flows and molecule velocity; satellite-to-core size ratio considerations; model for self-reconfiguring active molecules accounting for molecule dynamics, intramolecule interactions, and intermolecule interactions between neighbors; self-assembly of molecules comprising differently sized, 4.0 μm SiO_2_ and 0.5 μm PS, spheres (Figure S1); measured dielectric properties and polarizability of the 5.6 μm SiO_2_ and 0.7 μm PS particles of the main text (Figure S2); schematic of the control experiment with preassembled molecules comprising 5.6 μm SiO_2_ and 0.7 μm PS particles, instantaneous velocities, separation distance between silica and polystyrene units, mean squared angular displacement curves, and time scale for reorientation as a function of peak-to-peak voltage (Figure S3); time scale for reorientation as a function of satellite translational diffusivity for trimers, and time scale for reorientation as a function of number of satellites extracted from simulations (Figure S4); velocity of self-reconfiguring tetramers comprising 5.6 μm SiO_2_ and 0.7 μm PS particles as a function of outer opening angle between satellites (Figure S5); speed of a self-reconfiguring pentamer comprising 5.6 μm SiO_2_ and 0.7 μm PS particles as a function of time as it approaches, senses and avoids a neighboring active molecule, and kurtosis of the angular displacement distributions as a function of lag-time for molecules with varying numbers of satellites that undergo consecutive encounters with neighbors (Figure S6) (PDF)
Self-assembly and propulsion of reconfiguring active colloidal molecules comprising silica and polystyrene microparticles under an AC electric field (AVI)
Active colloidal molecules with dynamic orientational freedom, and reconfiguration-induced self-propulsion and reorientation at low area fraction (MP4)
Dynamic-reconfiguration-induced steering and avoidance in active colloidal molecules in the presence of neighbors at intermediate area fraction (AVI)
Collective dynamics in active molecules with dynamical orientational freedom at high area fraction without activity-induced clustering (AVI)

## §

John A. Paulson School of Engineering and Applied Sciences, Harvard University, Cambridge, MA 02138, USA

## ∥

Physics Department, Universitá di Roma La Sapienza, P.le Aldo Moro 2, 00185, Rome, Italy.

## ⊥

University of Bordeaux, CNRS, CRPP, UMR 5031, F-33600 Pessac, France.
