# Supplementary material for: Active Colloidal Molecules with Dynamic Configurational Freedom
Source: ACS Nano. 2025 Aug 11;19(32):29430–9. doi: 10.1021/acsnano.5c07142 (PMC12369011; doi:10.1021/acsnano.5c07142)
Supplement: Supplementary file 1 [file nn5c07142_si_001.pdf]

Supporting Information for: Active Colloidal Molecules with Dynamic Configurational Freedom

Stefania Ketzetzi,[†,‡] Lorenzo Caprini,[¶,§] Vivien Willems,[†,‖] Laura Alvarez,[†,‖] Hartmut Löwen,[¶] and Lucio Isa[*,†]

†*Laboratory for Soft Materials and Interfaces, Department of Materials, ETH Zürich, Switzerland.*

‡*Current address: John A. Paulson School of Engineering and Applied Sciences, Harvard University, Cambridge, MA 02138, USA.*

¶*Institut für Theoretische Physik II: Weiche Materie, Heinrich-Heine-Universität Düsseldorf, D-40225 Düsseldorf, Germany.*

§*Current address: Physics Department, Universitá di Roma La Sapienza, P.le Aldo Moro 2, 00185, Rome, Italy.*

‖*Current address: University of Bordeaux, CNRS, CRPP, UMR 5031, F-33600 Pessac, France.*

E-mail: lucio.isa@mat.ethz.ch

Description of supplementary videos

File Name: Video 1

Description: Self-assembly and propulsion of reconfiguring active colloidal molecules comprising 5.6 μm SiO_2 and 0.7 μm PS particles under an AC electric field. The SiO_2 particles constitute the molecules' core while the PS particles are the molecules' satellites. The movie is taken on an inverted microscope equipped with a 40x Zeiss LD Plan-NEOFLUAR (NA 0.6) air objective and additional 1.6x magnification (0.1 μm/px) at 10 fps. At the beginning, the AC field is off. After 100 frames, the AC field is turned on (1 kHz, 1 V_{pp}, where V_{pp} is the AC voltage peak-to-peak amplitude). Thereafter, the frequency is kept constant and the amplitude of the field increases every 100 frames by 1 V_{pp} consecutively (from 1 to 5 V_{pp}). At 2 V_{pp}, many molecules have formed. At 3 V_{pp}, all molecules have formed and start to show directionality in their motion with the PS particles at the back. Propulsion velocity increases with increasing applied field amplitude up to 5 V_{pp}.

File Name: Video 2

Description: Dynamic orientational freedom and reconfiguration-induced propulsion and reorientation at the single-molecule level driven by random fluctuations (AC field condition: 1 kHz, 6 V_{pp}) in the absence of interactions with neighbors (molecule area fraction $\lesssim 1\%$). Actively self-propelled colloidal trimer and tetramer with two and three PS satellites respectively, imaged with a 63x Zeiss LD Plan-NEOFLUAR air objective (NA 0.75) and additional 1.6x magnification (0.069 μm/px) at 10 fps. Driven by Brownian fluctuations, PS satellites translate along the surface of the SiO_2 cores. Asymmetric distribution of satellites with respect to the core leads to directed motion, while a symmetric distribution leads to slowing down and potential change in the direction of motion, depending on the instantaneous configuration of the full molecule. At that area fraction, encounters between molecules are rare and reorientation is predominantly driven by fluctuations.

File Name: Video 3

Description: Reconfiguration-induced self-avoidance in active colloidal molecules in the presence of neighbors (molecule area fraction $\approx 4\%$, AC field condition: 1 kHz, 6 V_{pp}). Imaged with a 40x Zeiss LD Plan-NEOFLUAR air objective (NA 0.6) (0.16 μm/px) at 10 fps. With increasing molecule area fraction, the self-propelled molecules are likely to encounter neighbors. Upon encounter, they spontaneously sense each other from a distance and actively respond by changing propulsion direction, exhibiting self-steering capabilities.

File Name: Video 4

Description: Collective dynamics in self-reconfiguring active molecules (molecule area fraction $\approx 11\%$, AC field condition: 1 kHz, 6 V_{pp}). Imaged with a 40x Zeiss LD Plan-NEOFLUAR air objective (NA 0.6, 0.16 μm/px) at 10 fps. Self-avoidance hinders dynamic clustering at higher area fractions and the ensemble remains fluid, distinctly from the activity-induced clustering predominantly observed in the active colloids literature already at $\approx 3\%$ area fraction.

Calculation of electrohydrodynamic flows and molecule velocity

As discussed in the main text, our colloidal molecules propel themselves relative to the electrode due to the asymmetry in the electrohydrodynamic (EHD) flow around the SiO_2 particles caused by the PS particles' self-assembly. The velocity of a dimer molecule, V_1, comprising one SiO_2 core particle and one PS satellite particle can be obtained in first order approximation as a linear combination of the EHD flow velocities U_i generated around each particle "i", evaluated as a function of distance, r, away from each particle surface[1,2] as:

$$U_i = \frac{C\ (K_i^{'} + K_i^{''})\ \bar{\omega}}{\eta\ (1 +\ \bar{\omega}^2)} \frac{3\ (r/R_i)}{2\ [1 + (r + R_i)^2]^{5/2}} \tag{1}$$

$$V_1 = \frac{U_{SiO_2}\ R_{PS} + U_{PS}\ R_{SiO_2}}{R_{PS} + R_{SiO_2}} \tag{2}$$

with η the medium viscosity, R_i the particle radius, $r = R_{PS} + R_{SiO_2}$, $C = \beta\varepsilon_m\varepsilon_0 H(V_{pp}/2H)^2$, β a constant prefactor used here as a single fit parameter, ε_m the solvent relative permittivity, ε_0 the vacuum permittivity, $\bar{\omega}$ the normalized angular frequency $\bar{\omega} = \omega H/\kappa D$ with $\omega = 2\pi f$ and f the frequency of the applied electric field, κ the inverse Debye length and D the ion diffusivity.

K' and K'' are the real and imaginary part of the Clausius-Mosotti factor $K^* = K' + jK''$[3] with $j = \sqrt{-1}$. They play a key role in U_i as they determine the sign of the EHD flows, as well as contributing to their magnitude. The Clausius-Mossotti factor in fact describes the polarizability of a particle suspended in a fluid and it dictates the magnitude and sign of the induced dipole moment and therefore the distortion of the fluid flow of a charged particle under an AC electric field.[3,4] In particular:

$$K' = \frac{\omega^2\varepsilon_0^2(\varepsilon_p' - \varepsilon_m')(\varepsilon_p' + 2\varepsilon_m') + (\sigma_p' - \sigma_m')(\sigma_p' + 2\sigma_m')}{\omega^2\varepsilon_0^2(\varepsilon_p' + 2\varepsilon_m')^2 + 2(\sigma_p' + 2\sigma_m')^2} \tag{3}$$

$$K'' = \frac{\omega\varepsilon_0(\varepsilon_p' - \varepsilon_m')(\sigma_p' + 2\sigma_m') - \varepsilon_0(\varepsilon_p' + 2\varepsilon_m')(\sigma_p' - \sigma_m')}{\omega^2\varepsilon_0^2(\varepsilon_p' + 2\varepsilon_m')^2 + 2(\sigma_p' + 2\sigma_m')^2} \tag{4}$$

with $\varepsilon_p^{'}$ and $\varepsilon_p^{'}$ the real part of relative particle and medium permittivity, $\sigma_p^{'}$ and $\sigma_m^{'}$ the real part of particle and medium conductivity. We show the ε_p' and σ_p' for both particle types in Figure S2a. These values were obtained from renormalization of dielectric spectroscopy measurements of the effective $\varepsilon_{eff}^{'}$ and $\sigma_{eff}^{'}$ values of each individual particle suspension as a function of frequency (10 to 10^6 Hz) at temperature 24° (Novocontrol high-resolution dielectric analyzer Alpha-A). For this, we considered Maxwell-Wagner-Sillars' theory,[5] where the complex dielectric function of a heterogeneous mixture of spherical particles relates to the dielectric properties of its components as:

$$(\varepsilon_{eff}^* - \varepsilon_m^*)/(\varepsilon_{eff}^* + 2\varepsilon_m^*) = \phi(\varepsilon_p^* - \varepsilon_m^*)/(\varepsilon_p^* + 2\varepsilon_m^*) = \phi\ K^*, \tag{5}$$

with ϕ the volume fraction of the particles dispersed in the matrix medium (1 wt % for PS particles and 0.1 wt % for SiO_2 particles), K^* the Clausius-Mossotti factor of the particles in the suspension, and ε_{eff}^*, ε_p^*, ε_m^* the complex permittivity of the suspension, the particle and the medium, respectively.

Using the measured ε_p' and σ_p' values of Figure S2 we then calculate K_i' and K_i''. The behavior of K in Figure S2b already suggests that for low f values, i.e., 1 kHz regime, interactions via EHD flows dominate and dipolar interactions are negligible. This is due to strong ionic screening effects, as the surface ions follow the polarization timescale of the applied electric field. As f increases moderately to $>$ 20 kHz, ionic screening is largely reduced, such that there is a competition between EHD and dipole interactions. In the MHz regime, ions cannot follow the rapidly oscillating field, and the bulk material response dominates, with particle interactions described by dipole-dipole interactions.

Thus, the main interaction dominating our experiments performed at 1 kHz is based on the attractive EHD flows around the SiO_2 particles, bringing the PS towards it and causing their self-assembly by geometrically yet flexibly trapping the PS between the electrode and the equatorial plane of the SiO_2 particle.

Using the K_i' and K_i'' values of Figure S2b, we predict U_i for each particle of the dimer evaluated at a position r corresponding to the center of the other particle using Eq. 1. The calculated relative flow velocities, U_{SiO_2} and U_{PS}, are shown as insets in Figure S2c together with a schematic representation (not to scale). The calculation shows that the magnitude of the U_{SiO_2} flow velocity decreases with increasing frequency. The flow is pointing inwards in the direction perpendicular to the electrode, as evidenced by the negative sign. Note that the magnitude of U_{PS} is considerably lower. Finally, we combine the flow velocities for each particle to obtain the dimer velocity V_1 in the main panel of Figure S2c. This is obtained as a linear combination, see Eq. 2, and matches the experimentally measured instantaneous velocity of self-reconfiguring dimers, see Figure S2c below.

Satellite-to-core size ratio consideration

Simultaneously achieving both assembly and dynamical orientational freedom that leads to propulsion and flexible reorientation under the AC electric field depends on the satellite-to-core size ratio, as this controls the balance of electrohydrodynamic (EHD) forces, dipolar interactions, and Brownian motion. If small particles are outside the range (either too small or large relative to the core), the molecule will either fail to assemble or become too rigidly bound to propel and reorient dynamically within the experimental time window.

If satellites are too small, they will not be reliably trapped by the EHD flows or induced dipole attraction of the core particle, as the field-induced forces scale strongly with particle size (volume), whereas Brownian motion becomes more dominant as size decreases. For instance, the dielectrophoretic force in an AC field scales as $\sim D^3$ (with D the particle diameter), while Brownian diffusivity increases with decreasing size (1/D). Sufficiently small particles diffuse away faster than the EHD flow can carry and "bind" them into orbit and hence do not become "trapped".[6] In prior studies, small particles on the order of 10-20% of the big particle's diameter were successfully captured into clusters forming linear structures.[7] Below that (*i.e.*, $\ll$10%), the induced flows and forces become so weak that the small particle

either never gets pulled into the “bond” or is easily knocked out by random kicks. Based on the scaling laws and under moderate AC fields (1 kHz, 6 Vpp) as in our experiments, a satellite diameter on the order of one-tenth of the core is needed to meet this condition. Thus, polystyrene spheres smaller than 0.5 μm would not become “trapped” into the EHD orbital flow of a 5-6 μm silica core, and thereby not form molecules (the field-induced attraction would not overcome Brownian dispersion).

If the small particles are too large relative to the core, they acquire stronger induced dipoles in the AC field which leads to stronger repulsive forces between the satellite and the central particle (and between satellites themselves). The EHD “bonding” flow must overcome these repulsions. Experiments showed that beyond a certain size ratio, dielectric bonds no longer form reliably. For instance, when mixing 2.5 μm and 4 μm dielectric spheres no molecule formation was observed.[8] In addition, purely geometrically, if satellites are larger, fewer of them can assemble around the core. Crucially, when the core surface is saturated by large satellites, the assembly becomes symmetric and immobile, as the induced flows are balanced, and the molecules do not exhibit directed motion.

Finally, for a molecule to reconfigure dynamically, the satellite particles must be able to move around the core under the influence of the flow. A large satellite will instead lock into a fixed position, and the larger the satellite, the more the pair will behave like a rigid structure without orientational freedom or dynamic reorientation. Indeed, similarly sized particles experience similar induced forces[7] and tend to align symmetrically, forming *e.g.* linear chains along the field lines.[9] To retain multiple “bonds” and freedom of motion, the satellite must be no more than about 25-30% of the core’s diameter, or less to ensure flexibility in configuration.[8] Thus, if our polystyrene spheres were too large (approaching the microns size of the silica), molecules would effectively start behaving like fixed assemblies with a permanent dipole.

Model for self-reconfiguring active molecules

An active self-reconfiguring molecule is a many-particle colloidal object in solution consisting of a core, i.e. a big colloidal particle with radius R_c, and N satellites, i.e. small colloidal particles with radius R_s. Satellites assemble around the core and are free to orbit along its longitudinal axis. Thus, once a molecule with N satellites is formed, asymmetric configurations in satellite arrangement can be observed. This molecule is considered active since it shows a swimming motion along the asymmetric axis determined by the relative position of satellites compared to the core.

Prior to molecule formation, core and satellite particles behave as passive objects, being symmetric colloids immersed in a solution that satisfies the Einstein relation. As such, they can be described by overdamped dynamics for their position, governed by the diffusion coefficient D given by:

$$D_\alpha = \frac{k_B T}{6\pi\eta R_\alpha}, \tag{6}$$

with k_B the Boltzmann constant, T the room temperature, and η the water dynamic viscosity. The term R_α represents the particle radius; $\alpha = c, s$ for cores and satellites, respectively.

Molecule dynamics. Since experiments show quasi-two-dimensional motion for the active molecules, here, we neglect the third dimension and restrict the dynamics to two dimensions. The dynamics of the system is determined by overdamped equations of motion for core and satellites mutually interacting through a potential. The experimentally observed active motion is generated by a non-conservative force that cannot be obtained from a potential. The dynamics for the core particle position $\mathbf{x}_{c_j}$ and satellite particle positions $\mathbf{x}_i$ read

$$\dot{\mathbf{x}}_{c_j} = \sqrt{2D_c}\xi_{c_j} + \frac{\mathbf{F}_{c_j}}{\gamma} + V_1\mathbf{n}_j \tag{7a}$$

$$\dot{\mathbf{x}}_i = \sqrt{2D_s}\xi_i + \frac{\mathbf{F}_i}{\gamma} + \frac{\mathbf{F}_i^r}{\gamma} + V_1\mathbf{n}_j\,, \tag{7b}$$

where the index $i = 1, ..., N$ identifies the i-th satellite of an active molecule consisting of a

core and N satellites, while the index $j = 1, ..., n$ denotes the j-th core particle and therefore j-th molecule. In general, we use the pedex c to denote variables and forces characterizing the core particle. The terms ξ_{c_j} and ξ_i are independent white noises with zero average and unit variance. In this dynamics, the constant γ represents the effective friction coefficient due to the water solution so that $\mathbf{F}_{c_j}$ and $\mathbf{F}_i$ are the forces acting on core and satellites, respectively. Finally, $V_1 \mathbf{n}_j$ corresponds to the active velocity of the molecule and, thus, governs the dynamics of both cores and satellites.

Intra-molecule interactions. As seen in our experiments as well as the flow calculation above and Figure S2 below, when satellite and core particles are close they attract each other and form a stable molecule. This mechanism is modeled by considering an attracting force $\mathbf{F}_i$ between the satellite i and the core particle j. Consequently, the force $\mathbf{F}_{c_j}$ acting on the core is due to the contribution of all the molecule satellites and is given by $\mathbf{F}_{c_j} = -\sum_{i=1}^{i=N} \mathbf{F}_i$. This attraction is due to a Lennard-Jones potential $\mathbf{F}_i = -\nabla_i U_{LJ}(|\mathbf{x}_i - \mathbf{x}_c|)$, which constrains the satellite center of mass position to be at distance $\sigma_{cs} = R_c + R_s$ from the center of mass position of the core particle. Explicitly, $U_{LJ}(|\mathbf{x}_i - \mathbf{x}_c|)$ can be expressed as:

$$U_{LJ}(r) = 4\varepsilon_{cs} \left[\left(\frac{\sigma_{cs}}{r} \right)^{12} - \left(\frac{\sigma_{cs}}{r} \right)^{6} \right], \quad r < 3\sigma_{cs} \tag{8}$$

and zero otherwise for $r \geq 3\sigma_{cs}$. This potential is cut at distance $r = 3\sigma_{cs}$ and additionally shifted by the constant $-U_{LJ}(3\sigma_{cs})$ so that it is continuous in zero. The term ε_{cs} determines the typical energy scale of the potential which also fixes the potential barrier to observe the molecule breaking. Therefore, the molecule stability is guaranteed if thermal fluctuations are smaller compared to the potential barrier due to the attractive force $\mathbf{F}_{c_j}/\gamma$, with γ the effective friction coefficient of water. This implies that the molecule is stable if the following condition holds $D_s \ll \varepsilon_{cs}/\gamma$.

Additionally, satellites repel each other with an extra force $F_i^r = -\nabla_i U_{tot}^r$ due to a pure-repulsive potential $U_{tot}^r = \sum_{i<j} U^r(|\mathbf{x}_i - \mathbf{x}_j|)$. The shape of $U^r(r) = U^d(r) + U_w(r)$ consists of

a dipole-dipole repulsive potential and a Weeks-Chandler-Anderson repulsive potential. The first is defined as

$$U^{d}(r) = \varepsilon_{d}\left(\frac{\sigma_{s}}{r}\right)^{3}, \quad r < 5\sigma_{s} \tag{9}$$

and reads zero for $r > 5\sigma_s$, while the second is defined as

$$U_{w}(r) = 4\varepsilon_{w}\left[\left(\frac{\sigma_{s}}{r}\right)^{12} - \left(\frac{\sigma_{s}}{r}\right)^{6}\right], \quad r < 2^{1/6}\sigma_{s} \tag{10}$$

and zero otherwise. In addition, $\sigma_s = 2R_s$ is the satellite diameter, while ε_d and ε_w represent the typical energy scale of the dipole-dipole and WCA repulsive potentials, respectively. Finally, both potentials are shifted by irrelevant constants, $C^d = -U^d(5\sigma_s)$ and $C_w = -U_w(2^{1/6}\sigma_s)$, respectively, so that both are continuous functions.

Active velocity. The term $V_1\mathbf{n_j}$ models the experimentally observed effective driving velocity of the j-th molecule, which affects the evolution of the core and satellites. Specifically, the constant V_1 is the velocity scale observed in the simpler (dimer) molecule, consisting of a 5.6 μm core and a 0.7 μm satellite, and is chosen so that the measured dimer molecule velocity is reproduced by numerical simulations. The vector $\mathbf{n_j}$ identifies the direction of the active velocity. This is not a unit vector as it additively accounts for the contribution of each satellite. It is defined as $\mathbf{n}_j = \sum_{i=1}^{N} \mathbf{n}_{ij}$ with $\mathbf{n}_{ij}$ given by:

$$\mathbf{n}_{ij} = \theta(r_p - r_{ij})\frac{\mathbf{r}_{ij}}{r_{ij}^{3}}\sigma_{cs}^{2}\,. \tag{11}$$

Here, $\mathbf{r}_{ij} = \mathbf{x}_i - \mathbf{x}_{c_j}$ is the vector pointing between the core j and the satellite i while $r_{ij} = |\mathbf{r}_{ij}|$ is its modulus. The function θ selects satellites whose distance is smaller than r_p from the core. Each satellite contributes to the non-conservative force proportional to the effective velocity V_1, the typical swim velocity of the molecule, which is additionally modulated in space by a function of the distance. The choice of the force shape scaling as $1/r_{ij}^2$ is arbitrary and is chosen as in previous works.[10] The value of r_p is arbitrary and is chosen as $r_p = 2.5\sigma_{cs}$ so

that only satellites forming a molecule contribute to the molecule's active velocity. However, in the strong attraction limit considered in this work, $D_s \ll \epsilon_{cs}/\gamma$, the spatial modulation shape and the cutoff value are irrelevant.

Inter-molecule interactions. When two molecules swim towards each other, cores as well as cores and satellites of different molecules interact with each other. Here, we assume that these interactions are conservative and due to an effective potential generated by hydrodynamic interactions. A system with n molecules evolves with dynamics (7) complemented with the additional inter-molecular interactions. Core-core interactions are due to a long-range pure repulsive potential, core-satellite interactions are obtained from a long-range attractive potential, and satellite-satellite forces belonging to different molecules are negligible and simply give rise to extra repulsion.

Specifically, interactions between different molecules can be calculated from the total interacting potential U_{tot}^{inter} which is given by:

$$U_{tot}^{inter} = \sum_{j<l}^{n} U_{cc}^{inter}(|\mathbf{x}_{c_j} - \mathbf{x}_{c_l}|) + \sum_{i=1}^{N} \sum_{j=1}^{n} U_{cs}^{inter}(|\mathbf{x}_{c_j} - \mathbf{x}_i|) \,. \tag{12}$$

Here, $U_{cc}^{inter}(r)$ is the core-core repulsive potential which reads:

$$U_{cs}^{inter}(r) = -4\epsilon_{cs}^{inter} \left(\frac{\sigma_{cs}}{r}\right)^{0.5} \qquad r < 5\sigma_{cs} \tag{13}$$

and zero for $r > 5\sigma_{cs}$. The constant ϵ_{cs}^{inter} sets the energy scale of this interaction. The potential is cut at $5\sigma_{cs}$ and is shifted to be continuous by adding the constant $C_{cs}^{inter} = -U_{cs}^{inter}(5\sigma_{cs})$. In addition, $U_{cs}^{inter}(r)$ is the core-satellite attracting potential chosen as:

$$U_{cc}^{inter}(r) = -4\epsilon_{cc}^{inter} \left(\frac{\sigma_{cc}}{r}\right)^{0.5} \qquad r < 5\sigma_{c} \tag{14}$$

and zero for $r > 5\sigma_c$. The potential is cut at $5\sigma_c$ and shifted to be continuous by adding the

constant $C_{cc}^{inter} = -U_{cc}^{inter}(5\sigma_c)$. Finally, the constant ε_{cc}^{inter} determines the energy scale of this interaction. The total inter-molecule force acting on the satellite is given by: $\mathbf{F}_i^{inter} = \nabla_i U_{tot}^{inter}$ and the one acting on the core by: $\mathbf{F}_{c_j}^{inter} = \nabla_{c_j} U_{tot}^{inter}$. Here, ∇_i with $i = 1, ..., n$ denotes the vector derivative with respect to the satellite position, while ∇_{c_j} with $j = 1, ..., N$ represents the vector derivative with respect to the core position. Note that in this way the forces between cores and satellites of different molecules scale as $\sim 1/r^{1.5}$. Such a choice was inspired from the shape of the effective potential generated by hydrodynamic interactions.

Parameter choice. The parameters of the model are chosen according to the average experimental values, so that the ratio between the diameters of the two particles reads $\sigma_c/\sigma_s = 8$ with $\sigma_c = 5.6$. By using the Einstein relation with a room temperature of $T = 20^o$ C and the water dynamic viscosity η, the diffusion coefficient reads

$$D_c = 7.66 \times 10^{-2} \mu m^2 s^{-1} \tag{15a}$$

$$D_s = 6.13 \times 10^{-1} \mu m^2 s^{-1} \,. \tag{15b}$$

The active velocity of a dimer molecule with a core and a single satellite is given by:

$$V_1 = 3.4 \, \mu m s^{-1}. \tag{16}$$

As a result, we can estimate the effective Péclet number ruling the dynamics. The additional parameters for the numerical studies are:

$$\varepsilon_{cs} = 5 \tag{17a}$$

$$\varepsilon_d = 10 \tag{17b}$$

$$\varepsilon_w = 1 \tag{17c}$$

$$\varepsilon_{cc}^{inter} = 50 \tag{17d}$$

$$\varepsilon_{cs}^{inter} = 1 \,. \tag{17e}$$

References

1. Ma, F.; Yang, X.; Zhao, H.; Wu, N. Inducing Propulsion of Colloidal Dimers by Breaking the Symmetry in Electrohydrodynamic Flow. *Phys. Rev. Lett.* **2015**, *115*, 208302.

2. Ma, F.; Wang, S.; Wu, D. T.; Wu, N. Electric-field–induced assembly and propulsion of chiral colloidal clusters. *PNAS* **2015**, *112*, 6307–6312.

3. Pethig, R. *Dielectrophoresis*; John Wiley & Sons, Hoboken, NJ, 2017; Chapter 6, pp 119–144.

4. Shilov, V.; Delgado, A.; González-Caballero, F.; Horno, J.; López-García, J.; Grosse, C. Polarization of the Electrical Double Layer. Time Evolution after Application of an Electric Field. *J. Colloid Interf. Sci.* **2000**, *232*, 141–148.

5. Alvarez, L.; Fernandez-Rodriguez, M. A.; Alegria, A.; Arrese-Igor, S.; Zhao, K.; Kröger, M.; Isa, L. Reconfigurable artificial microswimmers with internal feedback. *Nature Communications* **2021**, *12*, 4762.

6. Ristenpart, W. D.; Aksay, I. A.; Saville, D. A. Electrohydrodynamic flow around a colloidal particle near an electrode with an oscillating potential. *Journal of Fluid Mechanics* **2007**, *575*, 83–109.

7. Bharti, B.; Findenegg, G. H.; Velev, O. D. Co-Assembly of Oppositely Charged Particles into Linear Clusters and Chains of Controllable Length. *Sci Rep* **2012**, *2*, 1004.

8. Wang, Z.; Wang, Z.; Li, J.; Tian, C.; Wang, Y. Active colloidal molecules assembled via selective and directional bonds. *Nature Communications* **2020**, *11*, 2670.

9. Heatley, K. L.; Ma, F.; Wu, N. Colloidal molecules assembled from binary spheres under an AC electric field. *Soft Matter* **2017**, *13*, 436–444.

10. Schmidt, F.; Liebchen, B.; Löwen, H.; Volpe, G. Light-controlled assembly of active colloidal molecules. *J. Chem. Phys.* **2019**, *150*, 094905.

Supporting figures

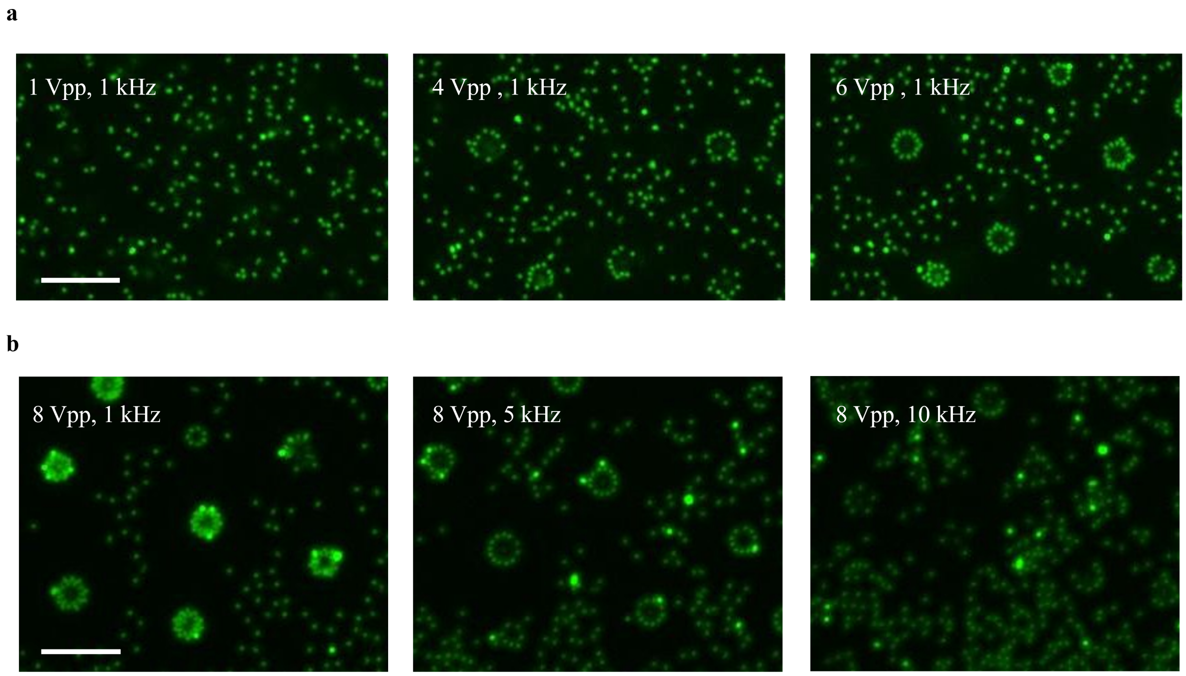

Figure S1: Self-assembly of colloidal molecules comprising 4.0 µm SiO_2 and 0.5 µm fluorescent PS colloids, i.e. different sizes but same size ratio as in the main text. a) Molecules are formed at frequency 1 kHz upon sequentially increasing, from left to right, the peak-to-peak amplitude from 1 to 4 to 6 V_{pp}. Here, only molecules with full valency were obtained due to the high concentration of PS particles in solution. Directed motion via dynamic reconfiguration is observed in this case for molecules that have a full ring plus additional PS particles as part of a second ring, with those outer PS particles diffusing along the second ring allowing for asymmetric configurations with respect to the core. b) Another way to disassemble the active molecules, other than decreasing the V_{pp} at 1 kHz, is to increase the frequency, as this modulates the interaction between SiO_2 and µm PS particles and thereby the electrohydrodynamic EHD flows. Here, the amplitude of the field is kept fixed at 8 V_{pp}, and the frequency is increased, from left to right, from 1 to 5 to 10 kHz, leading to disassembly. Scale bars are 10 µm. Data is taken in fluorescence mode on an Eclipse Ti2-e inverted microscope equipped with an S Plan Fluor ELWD 40x DIC N1 objective at 33 fps.

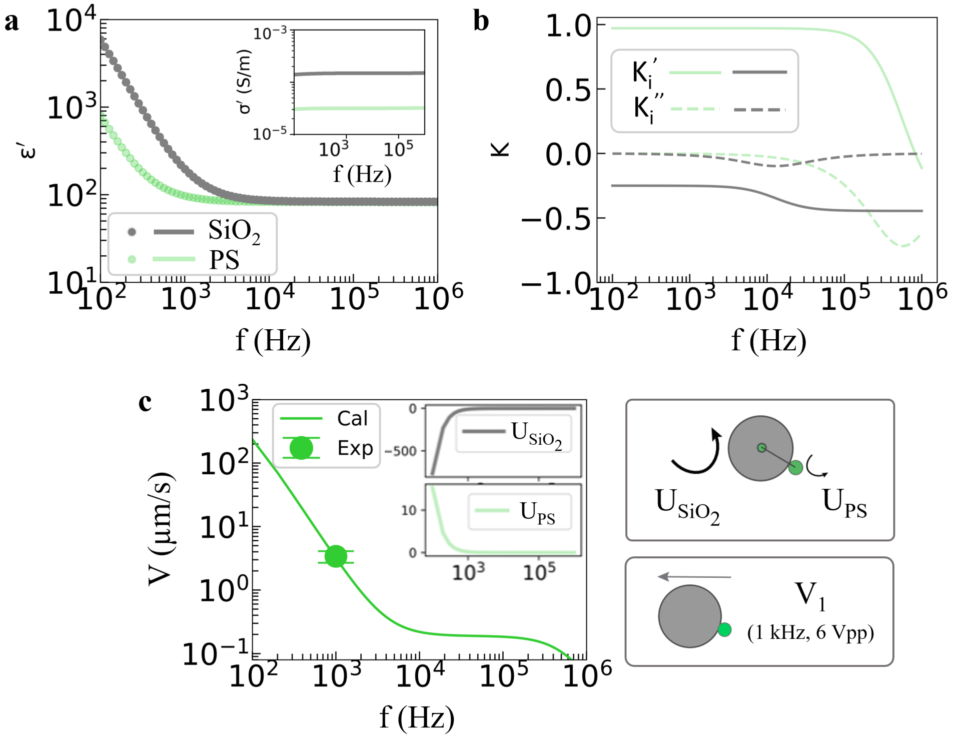

Figure S2: Dielectric properties and polarizability of the 5.6 μm SiO_2 and 0.7 μm PS colloids under study in the main text. a) Measured polarizability ε'_p (main) and surface conductivity σ'_p (inset) a function of frequency for each particle type at temperature 24°. b) Calculated Clausius-Mossotti (complex polarizability) factor as a function of frequency for each particle type. The values are obtained using the corresponding values in (a). c) Main panel: net molecule velocity V_1 as a function of frequency using Eq. 2, with the single fit parameters β_{PS} and β_{SiO_2} 1.3 and 1.6, respectively. Inset: theoretically predicted EHD flow velocities for each particle type, negative for the case of SiO_2 and positive for the PS, using the corresponding values in (b) and Eq. 1. Schematics indicate the direction of the positive (i.e., repulsive in the case of PS) and negative (i.e., attractive in the case of SiO_2) flows.

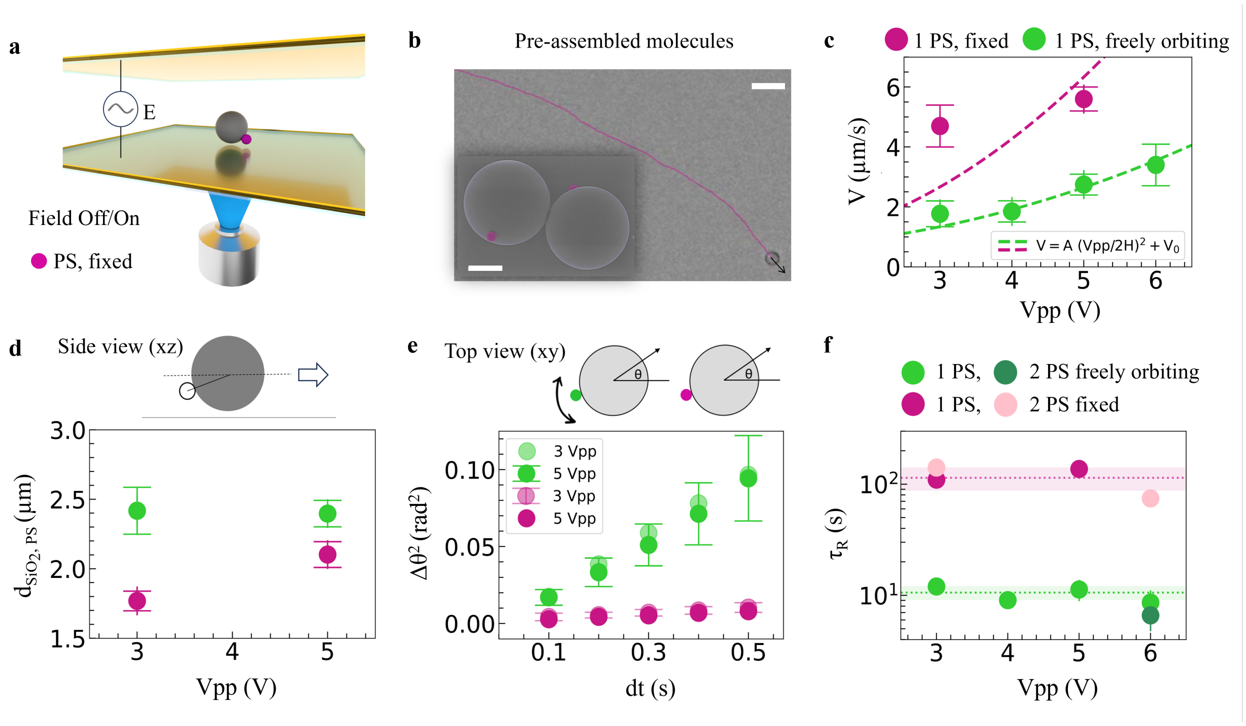

Figure S3: a) Schematic of the control experiment featuring pre-assembled molecules made from the same 5.6 μm SiO_2 and 0.7 μm PS colloids as the self-reconfiguring molecules of the manuscript, albeit with the PS fixed on the SiO_2 surface. b) Scanning electron microscopy image of two pre-assembled dimers (PS colloids false colored in magenta) and active trajectory of a pre-assembled dimer under the AC electric field at frequency 1 kHz. Scale bars are 2 and 10 μm, respectively. c) Average speeds $V = |\Delta\vec{r}|/\Delta t$ of molecules with one polystyrene particle, with $\Delta t = 0.1$ s as per the frame rate, both in the dynamically reconfiguring and pre-assembled cases as a function of peak-to-peak voltage. The dotted lines represent a least-squares fit with the expression $V = V_0 + A\ (V_{pp}/2H)^2$, where $V_0 = (0.6 \pm 0.3)$ μm/s is the average speed of a single core particle obtained from its Brownian displacements at $\Delta t = 0.1$ s and A is an experiment-dependent prefactor.[5] d) Measured center-to-center separation distance between the SiO_2 and PS particle forming the self-reconfiguring (green) and pre-assembled (magenta) dimer obtained directly from the particle positions indicating that molecules assume a different configuration with respect to the electrode. Errors denote standard deviations. The difference in configuration as a function of V_{pp} in the pre-assembled case suggests a difference in the prefactor A and might cause the deviation from the prediction in (c). The schematic shows a side view of the molecule-electrode geometry. e) Mean squared angular displacement MSAD curves of the core of self-reconfiguring (green) and pre-assembled (magenta) dimers as a function of lag-time dt; errors denote standard deviations. These are fitted to extract the rotational diffusion coefficients and corresponding timescales of reorientation as described in the manuscript. In the schematic, the angle θ represents the orientation of the velocity vector. f) Timescale for reorientation τ_R as a function of V_{pp} extracted from experiments on dimers and trimers with one and two PS particles, respectively. Shaded areas denote the standard deviation. Standard errors are included but are not visible due to the logarithmic scale.

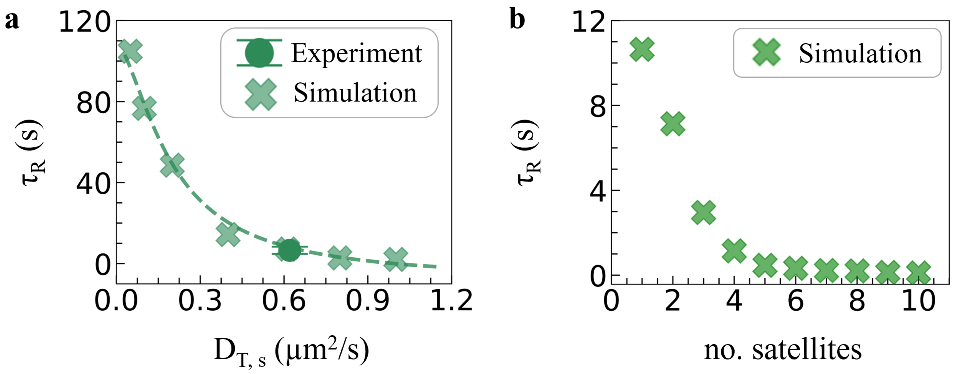

Figure S4: a) Timescale for reorientation τ_R as a function of satellite translational diffusivity $D_{T,s}$ for trimer molecules comprising a core and two satellite particles extracted from our simulations. Dashed line is a guide to the eye. b) Timescale for reorientation τ_R as a function of number of satellites extracted from simulations of self-reconfiguring molecules, assuming the same size and size ratio as in the experiments described in the main text

.

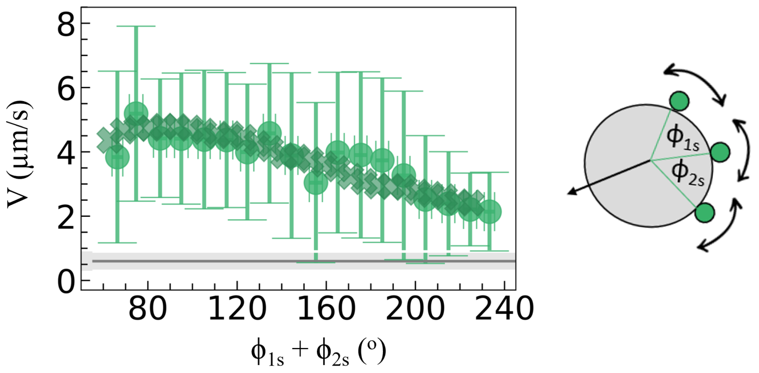

Figure S5: Instantaneous velocity V of dynamic self-reconfiguring tetramers, consisting of a 5.6 μm core and three 0.7 μm satellite particles, decreases as a function of the instantaneous outer opening angle between satellites, given by the sum of the two smallest angles measured between the three satellites (ϕ_{1s} and ϕ_{2s}, respectively), as illustrated in the schematic. Errors denote standard deviations. The gray line is the average speed, and the shaded area the standard deviation, of a single core particle obtained from its distribution of Brownian displacements at dt = 0.1 s ($V_0 = (0.6 \pm 0.3)$ μm/s).

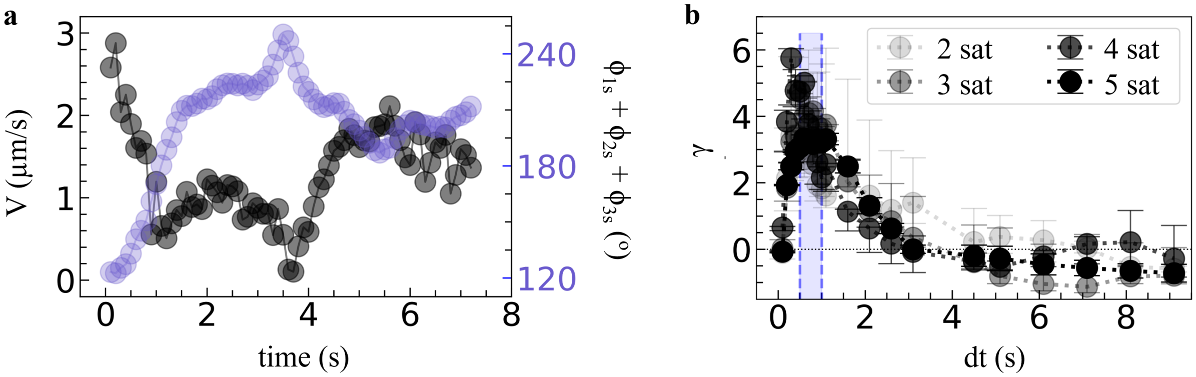

Figure S6: a) Speed V of a dynamically self-reconfiguring pentamer with 5.6 μm core and four 0.7 μm satellites as a function of time as it approaches, "collides", and self-avoids its neighboring active molecule. Secondary ψ-axis shows the outer opening angle between satellites, i.e. the sum of the three smallest instantaneous angles between satellites (ϕ_{1s}, ϕ_{2s}, and ϕ_{3s}, respectively) which follows the opposite trend, in agreement with the "sum rule" of the velocity. b) Kurtosis γ of the angular displacement distributions of cores as a function of lag-time dt for molecules with varying numbers of satellites that undergo consecutive "collisions". The height of the peak of the kurtosis increases with the number of collisions. The kurtosis peaks at ≈ 0.6 s commensurate with the duration of a collision (shaded region). Errors denote one standard deviation calculated from the 25 and 75% quartiles.
